# Influence of Aging and Surface Treatment on the Composite Bond Strength to Translucent 3Y-TZP Zirconia

**DOI:** 10.3290/j.jad.b3500591

**Published:** 2022-10-18

**Authors:** Nawal M. Moqbel, Majed Al-Akhali, Sebastian Wille, Matthias Kern

**Affiliations:** a Doctoral Student, Department of Prosthodontics, Propaedeutics and Dental Materials, School of Dentistry, Christian-Albrechts University at Kiel, Germany. Performed the experiments in partial fulfillment of requirements for her doctoral thesis, drafted the manuscript.; b Assistant Professor, Department of Prosthodontics, Propaedeutics and Dental Materials, School of Dentistry, Christian-Albrechts University at Kiel, Germany; Department of prosthodontics, School of Dentistry, Ibb University, Ibb, Yemen. Developed the idea, hypothesis and experimental design, contributed to the statistical evaluation, and contributed writing the manuscript.; c Research Scientist, Department of Prosthodontics, Propaedeutics and Dental Materials, School of Dentistry, Christian-Albrechts University at Kiel, Germany. Consulted on and contributed to the statistical evaluation, supervised the experiment, and reviewed and edited the manuscript.; d Professor and Chairman, Department of Prosthodontics, Propaedeutics and Dental Materials, School of Dentistry, Christian-Albrechts University at Kiel, Germany. Contributed to the experimental design, supervised the experiment, and reviewed and edited the manuscript.; * Nawal M. Moqbel and Majed Al-Akhali contributed equally to this work.

**Keywords:** translucent 3Y-TZP zirconia, aging, alumina-particle air abrasion, bonding, primer, luting composite.

## Abstract

**Purpose:**

The purpose of this study was to assess the effect of aging and alumina-particle air abrasion at different pressures on the bond strength of two luting composites to a translucent 3Y-TZP zirconia.

**Materials and Methods:**

Half of the 192 disk-shaped zirconia specimens were aged in an autoclave (group A) for 20 h (134°C, 2 bar), and the other half was not aged (group N). For each group, a different surface treatment was applied: as-sintered (group SIN), alumina-particle air abrasion either at 1 bar (group 1B) or at 2.5 bar (group 2.5B). Disks were bonded to Plexiglas tubes filled with composite resin using a phosphate monomer-based luting composite (group SA) or with a separate phosphate monomer containing primer before using a phosphate–monomer-free luting composite (group V5). All specimens were subjected to tensile bond strength testing (TBS) before and after thermocycling.

**Results:**

There were no statistically significant differences caused by autoclave aging for the test groups before thermocycling, except for the A-SIN-SA group, where the TBS decreased significantly. The variation of the alumina-particle air abrasion pressure showed no statistically significant effect, except in the N-1B-V5 group, where TBS was significantly lower than N-2.5B-V5. After thermocycling, the TBS of most groups decreased significantly. Specimens of the primer group, which were abraded at 1 bar, showed a significant decrease in TBS in comparison with alumina-particle air abrasion at 2.5 bar.

**Conclusion:**

Twenty hours of autoclave aging had almost no influence on the bond strength of the test groups. For the primer/resin bonding system, higher pressure during alumina-particle air abrasion might help obtain a higher and more durable bond strength to zirconia.

In recent years, the demand for esthetic all-ceramic restorations has increased, leading to the development of ceramic materials with improved mechanical properties and good esthetics. These ceramics are widely used in clinical applications, such as for posts, fixed dental prostheses, implant abutments and even adhesive resin-bonded fixed dental prostheses.^27^ Tetragonal, partially stabilized zirconia, known as conventional zirconia, was developed two decades ago, but its opacity is a problem, especially for anterior restorations. The translucency of zirconia is strongly related to its microstructure and chemical composition.^8^ The first generation of zirconia (3Y-TZP) was based on the conventional Y-TZP ceramic with increased sintering temperature and dwell time to produce a zirconia that still had high opacity but with good mechanical properties.^48,50^ The second generation of zirconia, also called translucent 3Y-TZP, consists of 3Y-TZP with a decreased grain size and amount of aluminum oxide. This small change in the material composition enhanced the light transmission and optical/esthetic properties while maintaining good long-term stability and high strength.^49^ The clinical outcome of this dental material greatly depends on its adhesion to natural teeth, alloys, and other dental materials.^35^

Minimally invasive esthetic dentistry requires the use of luting composite to bond a wide variety of indirect restorations to tooth structure. Luting composites have better properties than conventional luting agents, including higher strength, lower wear, and improved esthetics.^1,5,18,39^ Adhesive resins enhance the long-term survival of restored teeth through a strong adhesive bond between the dental restoration and the tooth structures.^23,54^

The addition of the phosphate-ester monomer 10-methacryloyloxydecyl dihydrogen phosphate (MDP) to the bonding agent (primer or adhesive resin) has been reported to produce a durable resin-to-zirconia bond, because chemical bonds are formed between MDP and zirconia^9,29,59^ and the wettability of the zirconia surface is increased.^20^ These findings have been confirmed in clinical trials.^45,53^ Self-adhesive composite cements are expected to bond to zirconia in the same way as other phosphate-based adhesive materials.^34^ MDP-based primers are available to promote the chemical adhesion of resin materials to the hydroxyl group present on the zirconia surface and to increase surface wettability.^20,62^ Surface treatments have been developed that either replace classic methods with new surface roughening procedures or combine them with other methods such as laser treatments or surface modifications that induce chemical bonding between zirconia and luting composite.^54^ A strong and durable bond to dental zirconia can be achieved through adequate cleaning of the bonding surfaces, micromechanical roughness created by alumina-particle air abrasion, or chemical activation using phosphate monomers such as MDP-based primers and/or luting composites.^23,40,54^

Alumina-particle air abrasion is an excellent procedure to increase the adhesion of luting composites to zirconia ceramics.^44,57^ Alumina-particle air abrasion followed by an appropriate chemical bonding process has been reported to result in long-term, durable bonding to zirconia.^29,41,58^ In addition, previous studies have reported that MDP-containing luting composites create a stable and strong bond to alumina-particle air-abraded zirconia that survives thermocycling.^29,33^ These findings have been supported by systematic reviews evaluating both in-vitro and clinical studies; these reveal strong clinical evidence that alumina-particle air abrasion with alumina particles at a moderate pressure (up to 2.5 bar) in combination with a phosphate–monomer-based primer and/or luting composite provides long-term durable bonding to zirconia ceramics under the stresses of oral conditions.^7,24^

Aging or low-temperature degradation (LTD) of zirconia is a process that can occur experimentally^10,63^ or in the oral environment by direct exposure to different stimuli, such as oral masticatory forces, the effect of water, pH changes, and temperature fluctuations,^10,11,14,21^ indicating that environmental conditions could accelerate LTD in zirconia ceramics. In addition to evaluating the adhesion to newly processed zirconia as received from the dental laboratory, it is also clinically relevant to evaluate adhesion to aged zirconia. It may be necessary to repair zirconia restorations with fractured veneers that have been exposed to the oral environment for a long time. The influence of aging and surface treatment on translucent 3Y-TZP zirconia has not been sufficiently investigated. Aging zirconia in an autoclave for 5 h at 134°C under 2-bar pressure has been reported to reduce microtensile bond strength after 24 h of storage when the zirconia specimens were bonded with the luting composite RelyX Unicem (3M Oral Care; St Paul, MN, USA). However, these specimens were not subjected to artificial aging with thermocycling, so long-term bond strength was not evaluated.^38^

In order to evaluate the bonding durability to translucent 3Y-TZP zirconia, the current study investigated the effect of different surface conditioning methods, including aging and alumina-particle air abrasion at varied pressures and priming, on the long-term resin bond strength to highly translucent zirconia. The null hypotheses of the current study were that no influence of (1) aging zirconia, (2) using different pressures during alumina-particle air abrasion, and (3) using different adhesive luting systems on bonding durability to translucent 3Y-TZP zirconia.

## MATERIALS AND METHODS

A translucent 3Y-TZP dental zirconia (Katana HT10, Kuraray Noritake; Tokyo, Japan) was used in this study. One hundred ninety-two disk-shaped specimens were cut and sintered in a sintering oven (Nabertherm; Bremen, Germany) according to the manufacturer’s instructions (1500°C; holding time: 2 h) to obtain fully sintered zirconia disks with a final diameter of 8 mm and a thickness of 3.2 mm. The underside of each disk was polished with rotary carbide paper to 600 grit. The specimens were then divided into two main groups according to aging: aged (group A) and non-aged (group N).

### Aging

Aging was performed after sintering on half of the zirconia disks (n = 96) according to ISO standard 13356 at 134°C under 2 bar for a period of 20 h in an autoclave (CS, WEBECO; Bad Schwartau, Germany). Subsequently, the specimens were cleaned in an ultrasonic bath with 99% isopropanol for 3 min. This aging protocol was used in this study, because previous studies have shown that aging at 134°C under 2 bar for 20 h promotes extensive t→m phase transformation.^10,21^ Thus, the aging performed in this study was considered sufficient to observe significant changes induced by different surface treatments.

### Surface Treatment and Bonding

For each main group, a different surface treatment was applied: no surface treatment after sintering (group SIN), alumina-particle air abrasion either with 1 bar (group 1B), or 2.5 bar (group 2.5B) using a spot-blasting unit (P-G 400 K Spot Fine Blasting Unit, Harnish+Rieth; Winterbach, Germany). Air abrasion was performed with 50-µm Al_2_O_3_ particles for 15 s at a distance of 10 mm with nozzle motion in both the horizontal and vertical directions. Subsequently, all specimens were cleaned in an ultrasonic bath with 99% isopropanol for 3 min. Three disks were selected from each group to be examined using XRD to evaluate the phase transformation of zirconia as described above.

Plexiglas tubes with a standard diameter of 3.2 mm were filled with self-polymerizing restorative composite resin (Clearfil FII New Bond, Kuraray Noritake; Tokyo, Japan) (n = 16/group). The self-polymerizing restorative composite resin polymerized in 10 min. Each group was divided into 2 subgroups (n = 16/subgroup) according to the luting composite used: group V5 (Panavia V5; Kuraray Noritake) and group SA (Panavia SA Plus Automix; Kuraray Noritake). For group V5, disks were conditioned with a universal ceramic primer (Clearfil Ceramic Primer Plus; Kuraray Noritake). A coating of primer was applied with a brush to the bonding surface for 10 s and allowed to dry. The entire bonding surface was then dried with a mild, oil-free air stream. Then, the dual-curing luting composite site was applied. For group SA, the specimens were bonded directly with the dual-curing adhesive resin.

**Fig 1:**
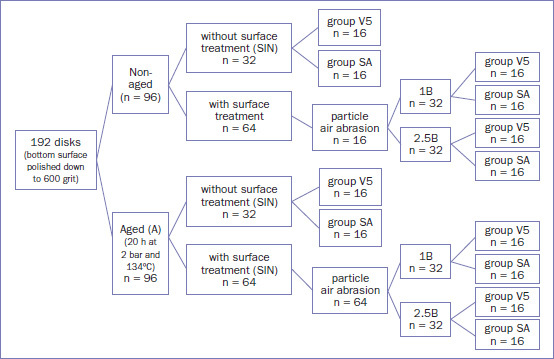
Schematic representation of the study design for evaluating tensile bond strength.

The filled tubes were then bonded to the zirconia surfaces using an alignment apparatus under a load of 7.4 N.^4,32,60^ The apparatus ensured that the tube axis was perpendicular to the zirconia bonding surface. Excess luting composite was removed with a sponge pellet, and oxygen-blocking gel (Oxyguard II; Kuraray Noritake, Okayama, Japan) was applied at the bonding margins. The margins were light polymerized for 20 s from two opposite sides using an LED curing unit (SmartLite PS, Dentsply Sirona; Konstanz, Germany) with a light intensity of 900-1200 mW/cm^2^, followed by additional polymerization for 90 s in a light-polymerizing unit (Heraflash, Heraeus Kulzer; Hanau, Germany). The study design is illustrated in Fig 1.

Eight specimens from each group were stored in a distilled water bath at 37°C for 3 days without thermocycling to record the initial bond strength. The remaining 8 specimens were stored in the water bath at 37°C for 150 days, interrupted by thermocycling between 5°C and 55°C in distilled water with a dwell time of 30 s for 37,500 cycles before measuring the tensile bond strength.^2,25,60,61^ All the materials used and their compositions are listed in Table 1.

**Table 1:** Material compositions

Name		Lot No.	Composition	Manufacturer
Katana (HT10)		DLUTX/DMRFH	(ZrO_2_ + HfO_2_ + Y_2_O_3_) > 99.0%, yttrium oxide (Y_2_O_3_) > 4.5–≤6.0%, hafnium oxide (HfO_2_) ≤5.0% and other oxides ≤1.0%	Kuraray Noritake, Tokyo, Japan
Clearfil FII New Bond	Base paste	2F0016	Bisphenol A diglycidylmethacrylate <18%, hydrophobic aliphatic dimethacrylate, silanated silica filler, colloidal silica, accelerators, pigments	
	Catalyst past	2A0015	Bisphenol A diglycidylmethacrylate 5-25%, triethylene glycol dimethacrylate <7%, silanated silica filler, colloidal silica, catalysts, pigments	
Panavia V5		990010	Bisphenol A diglycidylmethacrylate 5-15%, triethylene glycol dimethacrylate <5%, silanated barium glass filler, silanated fluoroalminosilicate glass filler, colloidal silica, surface-treated aluminum-oxide filler, hydrophobic aromatic dimethacrylate, hydrophilic aliphatic dimethacrylate, dl- camphorquinone, initiators, accelerators and pigments	
Clearfil Ceramic Primer Plus		9B0006	Ethanol >80%, 3-trimethoxysilylpropyl methacrylate <5% and 10-methacryloyloxydecyl dihydrogen phosphate (MDP)	
Panavia SA Plus		7U0058	MDP, Bis-GMA, TEG-DMA, hydrophobic aromatic dimethacrylate, HEMA, silanated barium glass filler, silanated colloidal silica, dl-camphorquinone, peroxide, catalysts, pigments, hydrophobic aliphatic dimethacrylate, surface treated sodium fluoride, accelerators	
Oxyguard II		1B0039	Glycerol 50-70% , polyethyleneglycol, catalysts, accelerators, dyes	


### Phase Analysis

The percentage of monoclinic and tetragonal phases on the zirconia surface after aging and the different surface treatments was determined by x-ray diffraction (Seifert XRD 3000 PTS, GE; Munich, Germany). Three specimens were selected from each group, and each was placed in the holder of the diffractometer and subjected to Cu Kα radiation. The spectrum was recorded within the range of 20°–40°, with a step size of 0.04°, and with a scan time of 10 s per step. Voltage and current were set to 40 kV and 40 mA, respectively. Analysis software (Peakfit v4.12, Systat Software; Erkrath, Germany) was used to evaluate the integrated intensities of the relevant peaks of the diffraction patterns.

The proportion of monoclinic phase (X_m_) was calculated using the method developed by Garvie and Nicholson, as follows:^15^

X_m_ = (I_M(111)_ + I_M(111-)_) / (I_M(111)_ + I_M(111-)_ + I_T(111)_),

where M_(111)_ and M_(111-)_ represent the intensities of the monoclinic peaks and T_(111)_ indicates the intensity of the tetragonal peak. I_T_ and I_M_ represent the integrated area under the tetragonal (111) and monoclinic (111) peaks as well as monoclinic (111- ) peaks around 30º, 31º, and 28º, respectively. The volumetric fraction V_m_ was calculated according to Torayra and Yoshimura, as follows:^56^

V_m_ = (1.311*X_m_) / (1+0.311*X_m_)

### Tensile Bond Strength and Failure Mode

After storage under the different conditions, the tensile bond strength was determined with a universal testing machine (Zwick Z010, ZwickRoell Group; Ulm, Germany) at a cross-head speed of 2 mm/min using a self-aligning chain-loop attachment.^3,4,60,61^

The debonded surfaces of the zirconia specimens were examined with an optical macroscope (Wild Heerbrugg Leica Mikroskop M420; Heerbrugg, Switzerland) at 35X magnification. Debonded surfaces were classified as cohesive failure within the luting or composite resin, adhesive failure at the interface between the zirconia and luting composite, or mixed adhesive/cohesive failure mode. Failure areas of each mode were calculated and expressed as a percentage of the total bonding surface area for each test group. Representative specimens were sputter-coated with a conductive gold alloy layer approximately 30 nm thick, then examined in a scanning electron microscope (SEM, XL 30 CP, Philips; Kassel, Germany) with an acceleration voltage of 15 KeV.^4,60,61^

Data were collected and checked for normal distribution using the Shapiro-Wilk test. As the data were not normally distributed, statistical significance was tested with the Kruskal-Wallis test followed by pairwise comparison using the Mann-Whitney U-test (α = 0.05). A correction for multiple comparisons was performed with the Bonferroni-Holm method. All calculations were made with a statistical software program (IBM SPSS for Windows; Version 20.0, IBM SPSS; Armonk, NY, USA).

## RESULTS

### Aging and Phase Analysis

XRD showed that both aging and different surface treatments resulted in different amounts of monoclinic phase. The highest monoclinic phase ratio (vol%) was found in groups A-SIN, A-1B, and A-2.5B, while the groups N-1B and N-2.5B showed a lower monoclinic phase ratio and group N-SIN exhibited no monoclinic phase (Table 2).

**Table 2:** Mean and standard deviation (SD) of the monoclinic phase ratio of zirconia measured by XRD

Group	Aging	Monoclinic phase ratio mean ± SD (vol%)
SIN	Non-aged (N)	0.0 ± 0.0
	Aged (A)	39.9 ± 0.7
1B	Non-aged (N)	7.5 ± 2.4
	Aged (A)	41.5 ± 0.3
2.5B	Non-aged (N)	10.4 ± 1.5
	Aged (N)	38.5 ± 2.8


### Tensile Bond Strength

**Fig 2:**
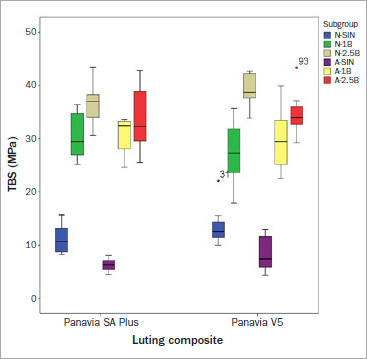
Boxplot of TBS after 3 days of storage.

**Fig 3:**
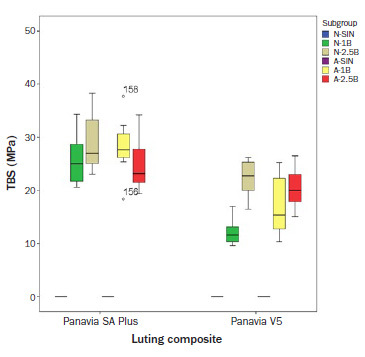
Boxplot of TBS after 150 days of storage with thermocycling.

The median tensile bond strength (TBS) for all groups is shown in Table 3. After 3 days of water storage, 20 h of aging showed no effect on the TBS, except in group A-SIN-SA, where TBS decreased significantly compared to the non-aged groups. Alumina-particle air abrasion caused a significant increase in TBS for all test groups. However, the different pressures used did not yield a statistically significant effect, except in group V5, where 1-bar pressure resulted in a statistically significantly lower TBS than did 2.5-bar pressure. Although most groups showed a significant decrease in TBS after long-term storage in water with thermocycling, group N-1B-SA showed no statistically significant decrease in TBS after thermocycling. In contrast, group V5, which was abraded at 1 bar pressure, showed a significant decrease in TBS after thermocycling compared with abrasion at 2.5 bar pressure. Figures 2 and 3 show boxplots for TBS after 3 days of storage and 150 days of storage with thermocycling.

**Table 3:** Tensile bond strength in MPa (medians, n = 8)

Main group	Group code	3 days	150 days
		Median	Median
Panavia SA, no aging (Gr factor 1)	N-SIN-SA	10.8 B,a,α	0.0 B,a,β
	N-1B-SA	29.5 A,a,α	25.1 A,ab,α
	N-2.5B-SA	37.0 A,a,α	27.0 A,a,β
Panavia V5, no aging (Gr factor 2)	N-SIN-V5	12.6 C,a,α	0.0 C,a,β
	N-1B-V5	27.3 B,a,α	11.7 B,c,β
	N-2.5B-V5	38.7 A,a,α	22.8 A,ab,β
Panavia SA, 20 h aging (Gr factor 3)	A-SIN-SA	6.4 B,b,α	0.0 B,a,β
	A-1B-SA	32.5 A,a,α	27.7 A,a,α
	A-2.5B-SA	32.4 A,a,α	23.2 A,ab,β
Panavia V5, 20 h aging (Gr factor 4)	A-SIN-V5	7.5 B,ab,α	0.0 B,a,β
	A-1B-V5	29.5 A,a,α	15.4 A,bc,β
	A-2.5B-V5	34.0 A,a,α	20.1 A,b,β

Statistically different medians (p ≤ 0.05) are indicated by different uppercase letters (within a column for the same grouping factor), or by different lowercase letters (within a column for the same surface treatment), or by different Greek letter (within a row).

**Fig 4:**
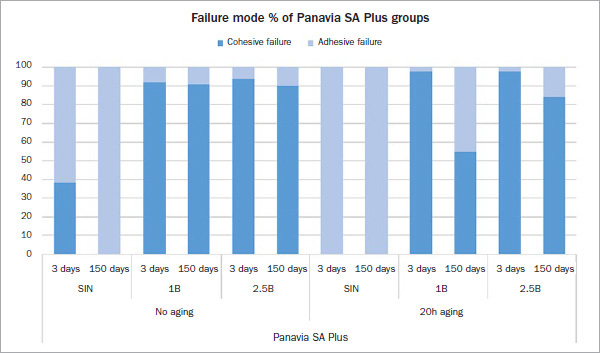
Type of bond failure mode as identified with a light microscope at 30X magnification and calculation in % of bonding area for all test groups bonded with Panavia SA Plus.

**Fig 5:**
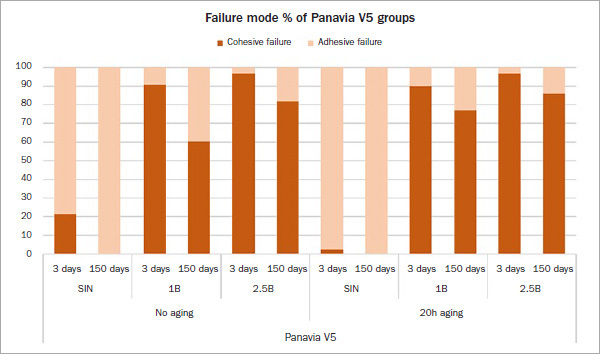
Type of bond failure mode as identified with a light microscope at 30X magnification and calculation in % of bonding area for all test groups bonded with Panavia V5.

**Fig 6:**
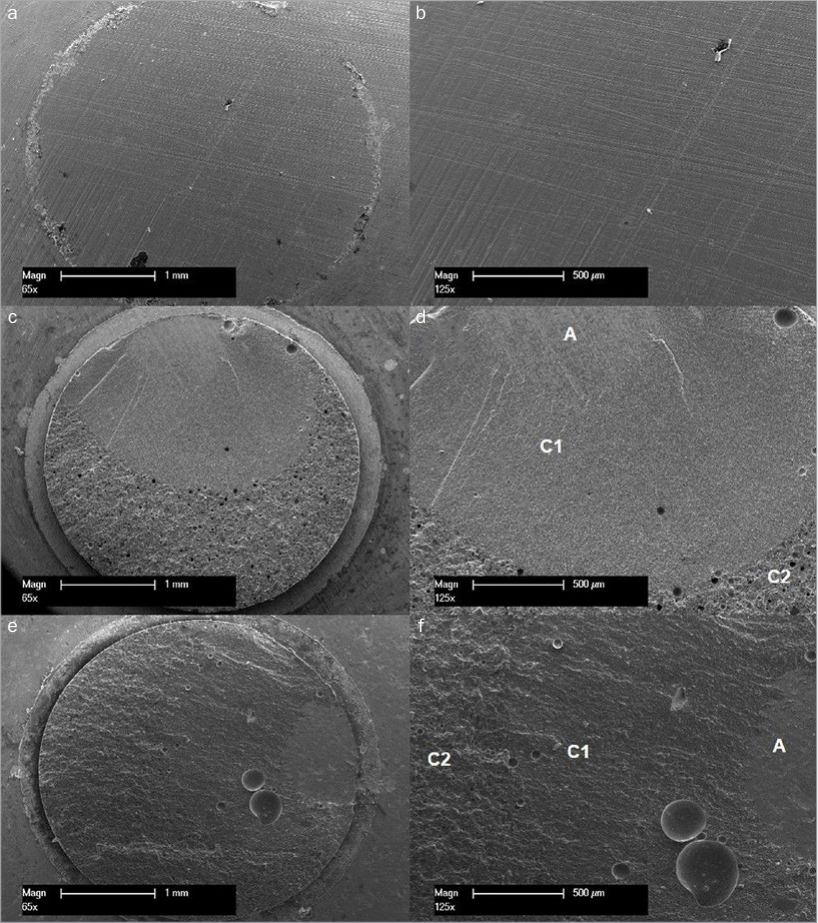
SEM of representative specimens of failure mode for the test groups; magnifications of 65X (a,c,e) and 125X (b,d,f) were used. (a) and (b) subgroup N-SIN-SA after 150 days of storage with thermocycling presents complete adhesive failure. (c) and (d) subgroup N-1B-V5 after 3 days of storage presents a predominantly cohesive failure mode. C1, cohesive failure in Panavia V5 luting composite; C2, cohesive failure in the tube resin. Only a small of area at the top failed adhesively (a). (e) and (f) subgroup N-1B-SA after 150 days of storage presents a predominantly cohesive failure mode; C1, cohesive failure in Panavia SA plus luting composite; C2, cohesive failure in the tube resin. Only a small of area on the right side failed adhesively (a).

The mode of failure of the test groups is illustrated in Figs 4 and 5. Most specimens tested after 3 days showed a predominantly cohesive failure within the luting composite and resin in the tube, except for groups that received no surface treatment. These groups showed almost exclusively adhesive failure. After thermocycling, groups A-1B-V5 and N-1B-SA shifted from cohesive to adhesive failure; in contrast, the other groups, except group SIN, still had predominantly cohesive failures. All group-SIN specimens debonded spontaneously during thermocycling in purely adhesive failure mode (Fig 6).

## DISCUSSION

Tensile bond strength testing was chosen because of its uniform stress distribution and to avoid inaccuracies resulting from complex forces occuring in shear bond strength tests.^12^ A microtensile bond test was not chosen to reduce technique sensitivity and exclude the micromovements and vibrations produced during specimen preparation.^43^ The weak point of the tensile bond strength test, ie, the perpendicular alignment of the composite resin tubes on the ceramic specimens, was solved by using a paralleling bonding device that ensured an exact 90° alignment on the ceramic surface.^55^

According to the ISO specification, thermocycling should be performed between 5° and 55°C, with a minimum of 500 cycles and a dwell time > 20 s. However, additional cycles or longer dwell times have been suggested to simulate more time in the oral cavity.^22,36,42^ In this study, the thermocycling protocol before TBS testing was adopted from several other studies on resin bonding to zirconia.^2,29,61^ This protocol was employed because it used the greatest number of cycles suggested in the literature (37,500 cycles), thus simulating more time in the oral cavity and ensuring complete water saturation of the bonded specimens.^2,13,29^

The aging protocol used in the current study has been reported to promote extensive t→m phase transformation,^10,21^ which should be sufficient to observe significant changes induced by different surface treatments. Low-temperature degradation initially occurs at the superficial grains, where water is incorporated into zirconia grains by filling oxygen vacancies and later spreads to the surface, increasing its roughness.^46,63^ Afterwards, LTD extends into the bulk of the material^63^ and negatively affects the density and mechanical properties of Y-TZP structures.^6,17,32^

The current study showed that 20 h of aging in an autoclave significantly decreased TBS only for group A-SIN-SA in comparison with the non-aged groups after 3 days of storage. A previous study^38^ reported that aging ZirCAD (Ivoclar Vivadent; Schaan, Liechtenstein) zirconia specimens for 5 h in an autoclave significantly decreased the bond strength after 24 h of storage in distilled water at 37°C without thermocycling. These specimens received no surface treatment and were bonded with RelyX Unicem (3M Oral Care) and conditioned with an MDP-based primer. This reduction in bond strength for the aged zirconia might be because of the surface alteration caused by the monoclinic phase on the zirconia surface, which might compromise the durability of the bond. These results were not considered clinically relevant, as TBS was reported after only a short period of storage without thermocycling.

Alumina-particle air abrasion of zirconia ceramic improves the bond strength of luting composite to zirconia and its durability.^26,60^ Increasing the surface roughness and bonding surface area leads to improved surface wettability and subsequently promotes bonding to zirconia.^28^ However, zirconia treated with high pressure alumina-particle air abrasion might develop surface cracks or fractures, and this might reduce its mechanical strength.^16,64^

In this study, alumina-particle air abrasion caused a significant increase in TBS for most test groups, consistent with previous studies.^44,51,57^ In addition, the different pressure had a statistical effect on the luting composite Panavia V5, where TBS was significantly higher after alumina-particle air abrasion with 2.5 bar than with 1 bar. In contrast, specimens bonded with Panavia SA plus were not affected by different pressures. A recent study^52^ reported that TBS of the specimens bonded with Panavia V5 increased with increasing pressure during alumina-particle air abrasion when evaluated after short-term water storage (1 day and 30 days without thermocycling). In contrast, using other bonding systems, low pressure of 0.5 bar during alumina-particle air abrasion effectively promoted the bonding of primer-containing adhesive luting systems.^25^ Also, another study reported that there were no significant differences in the bond strength after using different alumina-particle air abrasion pressures of 1, 2, 4 or 6 bar.^31^ Thus, it appears that the bond strength of some adhesives is affected more by varying alumina-particle air abrasion pressure than others.

The demand for adhesive bonding systems has increased with the use of esthetic ceramic materials in dentistry, as luting composites are generally superior to conventional luting cements in terms of strength, wear resistance, and esthetics.^5,18,38^ Self-adhesive luting composites have been introduced into the dental market to simplify bonding procedures of all-ceramic restorations. However, the used of luting composite with a separate primer showed more promising results for bonding to dentin and lithium-disilicate glass-ceramic.^37^ Furthermore, studies^20,54^ showed that pretreatment of surface zirconia with a 10-MDP-containing primer enhanced the bond strength and its durability. The current results showed a significant decrease in bond strength of most test groups after long-term storage in water with thermocycling, compared with only 3-day water storage, which is consistent with previous studies.^13,29,58^ A previous investigation^38^ reported that the aging of zirconia in an autoclave for 5 h vs 24-h water storage without thermocycling decreased the bond strength to zirconia significantly. However, the current study subjected specimens to 150-day water storage with additional thermocycling. The recorded reduction in bond strength to aged zirconia might be due to the plasticization effect of water on the resin matrix in long-term water storage with thermocycling,^19^ which leads to degradation within the luting composite itself, as has already been shown for other resin composites.^47^

The N-1B-SA and A-1B-SA groups showed no significant decrease in TBS. This finding is consistent with the results of a previous study,^65^ which reported that the presence of MDP in the luting composite improved its long-term bonding strength to zirconia. The current results confirm the clinical evidence that showed long-lasting bond strength of luting composites to zirconia when a phosphate–monomer-containing primer or luting composite was used in combination with alumina-particle air abrasion, even under moist and stressful oral conditions.^24^

The mode of failure after 3-day storage was predominantly cohesive in most of the test groups. The current results confirmed previous findings that adding a phosphate-ester monomer (10-MDP) promotes the bond strength of luting composite to zirconia.^30,60^ In addition, after long-term storage in water with thermocycling, most test groups still exhibited predominantly cohesive failures, with a statistically significant decrease in the bond strength. These findings concur with the results of previous studies^29,32,60^ in which 10-MDP-containing primers and luting composites were used, showing exclusively cohesive failures after 3-day storage and remaining nearly completely cohesive over 150 days of water storage with thermocycling. The observed decrease in bond strength might be due to the air bubbles in the luting composite, as detected during SEM examination, or from a reduction in cohesive strength of the luting composite caused by aging. In addition, as a result of the plasticization effect of water on the resin matrix,^19^ a certain degradation within the luting composite itself occurred, which has been reported for other composite resins.^47^ Moreover, groups which received no surface treatments debonded spontaneously during thermocycling in an almost exclusively adhesive failure mode. This confirms that the cleaning and roughening effect is essential to achieve durable bond strength of the adhesive to zirconia.^23,29,61^ Our results demonstrate that the 10-MDP monomer, either included in the luting composite or used as a separate primer, promotes a long-term durable bond to zirconia when combined with alumina-particle air abrasion treatment. However, the pressures tested led to different results depending on the type of bonding system used.

Limitations of the present study included its in-vitro design, which may not accurately simulate clinical conditions. Furthermore, no additional mechanical loading was applied to the bonded specimens in this study. In further studies, mechanical fatigue could be included along with thermal stresses to evaluate their combined influence.

## CONCLUSIONS

Twenty hours of aging in an autoclave does not affect the resin bond strength to translucent 3Y-TZP zirconia. For Panavia V5 luting composite (primer/resin system), higher pressure during alumina-particle air abrasion might help optimize the surface area of zirconia for bonding and obtain strong and durable bonding to zirconia.

## Clinical relevance:

Alumina-particle air abrasion improved TBS, while the pressures used yielded variable results depending on the type of bonding system employed.
